# Alternative Splicing of the Porcine Glycogen Synthase Kinase 3β (GSK-3β) Gene with Differential Expression Patterns and Regulatory Functions

**DOI:** 10.1371/journal.pone.0040250

**Published:** 2012-07-06

**Authors:** Linjie Wang, Bo Zuo, Dequan Xu, Zuqing Ren, Hongping Zhang, Xuewei Li, Minggang Lei, Yuanzhu Xiong

**Affiliations:** 1 Institute of Animal Genetics and Breeding, Sichuan Agricultural University, Ya’an, Sichuan, People’s Republic of China; 2 Key Laboratory of Swine Genetics and Breeding, Ministry of Agriculture & Key Laboratory of Agriculture Animal Genetics, Breeding and Reproduction of Ministry of Education, Huazhong Agricultural University, Wuhan, People’s Republic of China; Deakin School of Medicine, Australia

## Abstract

**Background:**

Glycogen synthase kinase 3 (GSK3α and GSK3β) are serine/threonine kinases involved in numerous cellular processes and diverse diseases including mood disorders, Alzheimer’s disease, diabetes, and cancer. However, in pigs, the information on GSK3 is very limited. Identification and characterization of pig GSK3 are not only important for pig genetic improvement, but also contribute to the understanding and development of porcine models for human disease prevention and treatment.

**Methodology:**

Five different isoforms of GSK3β were identified in porcine different tissues, in which three isoforms are novel. These isoforms had differential expression patterns in the fetal and adult of the porcine different tissues. The mRNA expression level of GSK3β isoforms was differentially regulated during the course of the insulin treatment, suggesting that different GSK3β isoforms may have different roles in insulin signaling pathway. Moreover, GSK3β5 had a different role on regulating the glycogen synthase activity, phosphorylation and the expression of porcine GYS1 and GYS2 gene compared to other GSK3β isoforms.

**Conclusions:**

We are the first to report five different isoforms of GSK3β identified from the porcine different tissues. Splice variants of GSK3β exhibit differential activity towards glycogen synthase. These results provide new insight into roles of the GSK3β on regulating glycogen metabolism.

## Introduction

The serine/threonine kinase glycogen synthase kinase 3 (GSK3) was first characterized for its role in glycogen metabolism with phosphorylating and inactivating the enzyme glycogen synthase [1], [2]. There are two mammalian GSK3 isoforms encoded by distinct genes: *GSK3α* and *GSK3β* [3]. The difference in size is due to a glycine-rich extension at the N-terminus of GSK3α. GSK3 performs an important role in several signalling pathways including IGF-1, Wnt and Hedgehog signal transduction, involved in the regulation of cell fate, embryonic development, protein synthesis, glycogen metabolism, mitosis and apoptosis [4]. In the absence of a Wnt signal, active GSK3 is present in a multiprotein complex that targets β-catenin for degradation via ubiquitin-mediated degradation [5], [6]. In Wnt stimulated cells, GSK3 phosphorylation of β-catenin is prevented, leading to accumulation of β-catenin and subsequent interaction with TCF/LEF transcription factors to trans-activate target genes [7]. IGF-1/Insulin may stimulate GS via the linear signaling cascade PI3K/Akt/GSK3β that leads to the phosphorylation of GSK3 and GSK3 kinase with inhibited activity. The inhibition of GSK3 by insulin results from its phosphorylation at Ser21 in GSK3α and Ser9 in GSK3β and that this is catalyzed by protein kinase B [8]. This inactivation leads to a decrease in phosphorylation of GS resulting in its activation and promotes glycogen synthesis [9].

The actions of GSK3 are also often regulated by the phosphorylation state of its substrates including cytoskeletal proteins, transcription factors and membrane receptors [10], [11]. Previous studies have shown that GSK3β has two alternatively splice variants (GSK3β1 and GSK3β2) [12], [13], and exhibits differential activity towards specific substrates. Recently studies show that GSK3β1 phosphorylates the microtubule-associated protein tau and MAP1B more effective in vitro than GSK3β2 [14]–[17], suggesting that different GSK3β isoforms could alter substrate specificity.

So far, most of the studies on GSK3 have been carried out in humans and mouse, few results have been reported on pigs. Although mouse has been used as human disease models, the pig is considered as an important experimental animal model of human disease, because pigs and humans share similar anatomical, physiological, and pathological characteristics. The knowledge of porcine GSK3, therefore, will also contribute to the understanding and development of porcine models for human disease (eg: Alzheimer’s disease and diabetes) prevention and treatment [18], [19].

Here we show that porcine GSK3β is multiple alternatively spliced in different tissues. The mRNA expression level of GSK3β isoforms is differentially regulated during the course of the insulin treatment. To further understand different GSK3β isoforms roles in glycogen metabolism, the expression level of porcine glycogen synthase gene and glycogen synthase activity were detected in pig kidney epithelial cells (PK15). The characterization of porcine GSK3β will undoubtedly help in further understanding its roles in mammals and development of porcine models for human disease prevention and treatment.

## Results

### Characterization of the Porcine GSK3α and GSK3β Gene

Analysis of the cDNA sequences of the porcine GSK3 revealed the following: (1) The deduced cDNA of porcine *GSK3α* consists of 2077 bp that contains an ORF of 1452 bp encoding a protein of 483 residues with a calculated molecular mass of 50.9 kDa and an isoelectric point (pI) of 9.04. It contains a 5′-untranslated region of 23 bp (5′-UTR) and a 3′-untranslated region of 602 bp (3′-UTR) with a putative polyadenylation signal AATAAA located at 2052 to 2057 bp. (2) The porcine *GSK3β* cDNA consists of 1517 bp; computer analysis revealed a 1263-bp ORF flanked by a 130-bp 5′-UTR and a 124-bp 3′-UTR. The porcine *GSK3β* gene is predicted to encode a polypeptide of 420 amino acids with a molecular mass of 46.8 kDa and a pI of 8.98. The sequences of porcine *GSK3α* and *GSK3β* were deposited in GenBank (GenBank accession no. HM214803 and JN387127).

Similar to their orthologous genes in both human and mouse, porcine GSK3α and GSK3β showed the highest homology toward their kinase domains (98% identity). The main structural differences between GSK3α and GSK3β isoforms lie in the N- and C-terminal regions. There is a glycine-rich extension at the N-terminal of GSK3α. Activities of GSK3 are positively regulated by phosphorylation of tyrosine residues 279 and 216 for α and β isoforms, respectively, and negatively regulated by N-terminal serine phosphorylation (residue 21 and 9 for α and β, respectively) [20].

### Identification of Multiple Alternative Transcripts of Porcine GSK3β

Previous studies have reported the identification of two forms of GSK3β in human and mouse brain, which in this article are referred to as GSK3β1 and GSK3β2. During the cloning of porcine *GSK3β* gene, sequencing of individual clones revealed three novel forms of *GSK3β* mRNA. The sequences for the cDNAs encoding porcine GSK3β1, 2, 3, 4 and 5 were obtained and are deposited in GenBank as JN387128-JN387132, respectively.

**Figure 1:**
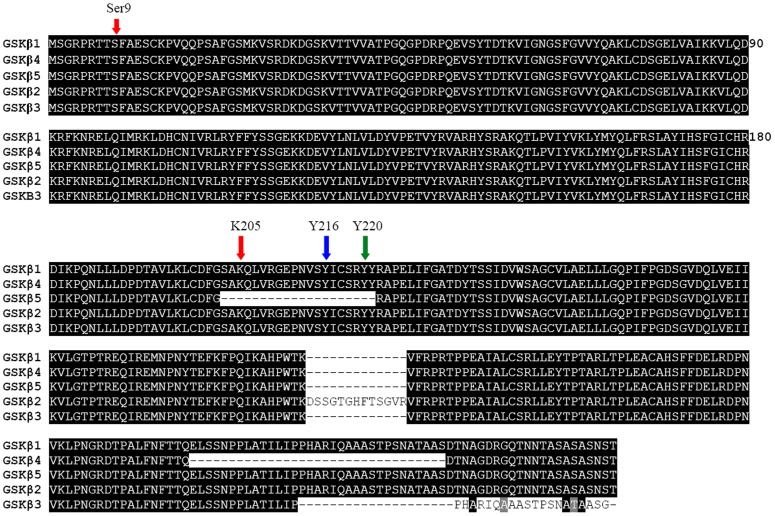
Alignment of amino acid sequences of the isoforms 1, 2, 3, 4, and 5 of GSK3β gene of porcine. GSK3β is spliced to five different kinds of mRNAs: GSK3β1, GSK3β2, GSK3β3, GSK3β4 and GSK3β5, which encode five proteins with 420, 433, 399, 387 and 400 amino acids, respectively. GSK3β2 have a 13 amino acid insert in the kinase domain. GSK3β3 and GSK3β4 contain the identical serine/threonine kinase domain but vary in their N-terminus. GSK3β5 lacks a 20 amino acid (203–222) in the kinase domain inculding three important phosphorylation site Lys 205, Tyr 216, Tyr 220.

**Figure 2:**
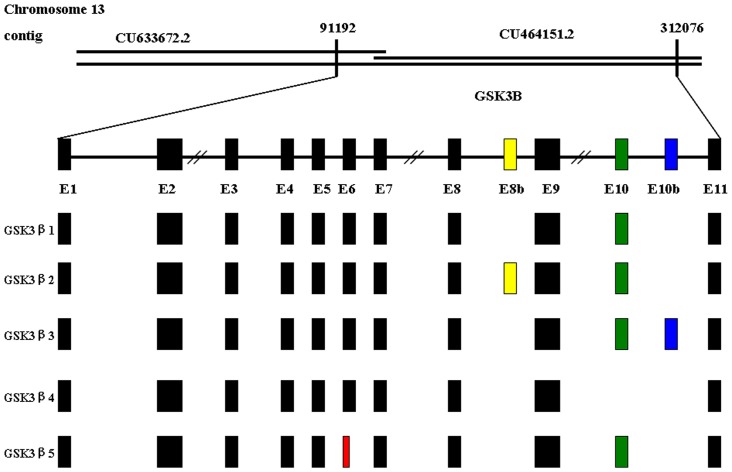
Schematic representation of the genomic and alternative splicing of porcine GSK3β. Line above shows the genomic contig from genome database. The exons and introns are represented by boxes and straight lines, respectively. GSK3β is spliced to five different kinds of mRNAs: GSK3β1, GSK3β2, GSK3β3, GSK3β4 and GSK3β5, respectively. GSK3β2 contained an additional exon8 b (yellow), GSK3β3 contained an additional exon10 b (blue), and GSK3β4 lacks exon10 (green) and GSK3β5 lacks 60 nucleotides in exon6 (red), resulting in a 20 amino acid extraction in the kinase domain. The sequences of porcine GSK3β1, 2, 3, 4 and 5 were deposited in GenBank (Accession No. JN387128 to JN387132).

These isoforms (GSK3β1, GSK3β2, GSK3β3, GSK3β4 and GSK3β5) encode predicted proteins with 420, 433, 399, 387 and 400 amino acids, respectively (Fig. 1). Interestingly, *GSK3β2* contained an additional 39 nucleotides insertion between exons 8 and 9, resulting in a 13 amino acid insert in the kinase domain. We call this minor exon, exon 8 b. This putative exon is surrounded by typical splice consensus sites. *GSK3β3* contained an additional 50 nucleotides insertion between exons10 and 11, named exon 10 b. The translational stop codon of *GSK3β3* exists in exon10. *GSK3β3* contains the whole kinase domain but lacks 21 amino acids at the N-terminus compared to isoform 1. *GSK3β4* lacks exon10 which encodes a region outside the catalytic domain, which is poorly conserved between species and between α and β isoforms. *GSK3β5* lacks 60 nucleotides in exon6, resulting in a 20 amino acid extraction in the kinase domain (Fig. 2).

### Genomic Structure of the Porcine GSK3β Gene

To get more information about the genomic structure of the GSK3β, we searched the pig nucleotide database by BLASTN, and found two overlapped contigs, which encode the GSK3β cDNAs (Fig. 2). The porcine GSK3β gene spans about 221 kb on chromosome 13. After comparing the genomic sequence with mRNA sequences, we found that there were thirteen exons, which were alternatively spliced to generate multiple GSK3β isoforms (Fig. 2). There were eleven exons for transcript of GSK3β1 and GSK3β5, twelve for GSK3β2 and GSK3β3, and ten for GSK3β4. When porcine GSK3β was compared with the GSK3β of human and mouse, an alignment of intron-exon junctions of GSK3β of these species was done, which showed that splicing sites (gt-ag) in all these introns of GSK3β were conserved in mammals. The GSK3β2 used a region of intron 8 sequence as its exon 8 b, and GSK3β3 used a region of intron 10 sequence as its exon 10 b (Fig. 2). The sequence of GSK3β3, GSK3β4 and GSK3β5 has not been cloned in other species.

### Differential Expression of GSK3β Isoforms in Fetal and Adult Porcine Tissues

**Figure 3:**
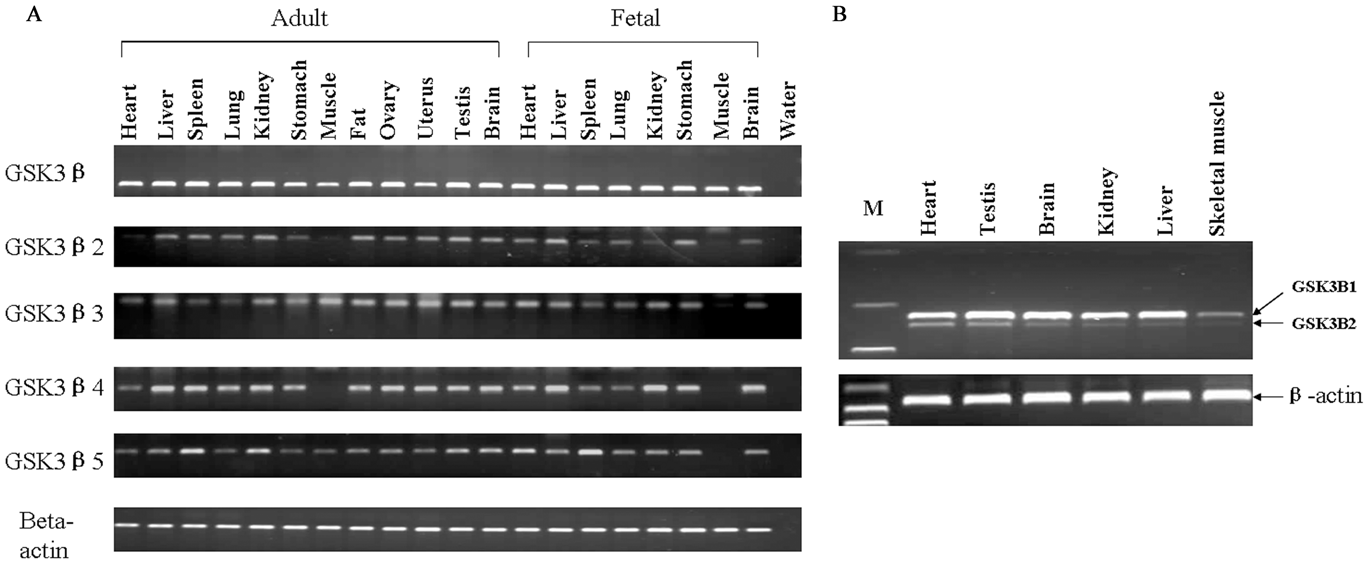
Expression analysis of GSK3β isoforms in porcine and mouse different tissues. (**A**) Expression analysis of porcine GSK3β isoforms in different fetal and adult tissues in Meishan pigs by RT-PCR and beta-actin was used as a control. PCR without cDNA template (water) served as negative controls. (**B**) Expression analysis of mouse GSK3β1 and GSK3β2 in different tissues by RT-PCR and beta-actin was used as a control.

To analyze expression patterns of these alternatively spliced GSK3β isoforms, the cDNAs synthesized from different fetal and adult tissues were analyzed by non-quantitative RT-PCR. As shown in Fig. 3A, five isoforms of the GSK3β expression are detectable in all tissues except for skeletal muscle. Porcine GSK3β4 mRNA was undetectable in adult skeletal muscle. In fetal skeletal muscle, the expression of GSK3β2, GSK3β3, GSK3β4 and GSK3β5 was absent. In order to determine whether three novel GSK3β isforms exists in mouse, we designed primer pairs between exon8 and exon10 for PCR, based on mouse GSK3β cDNA sequence information. After sequence analysis, we found that there was only two kinds of alternative splicing events of GSK3β (GSK3β1 and GSK3β2) in mouse different tissues (Fig. 3B). They are identical to the form reported previously [12], [13], and other GSK3β isoforms have not been found.

**Figure 4:**
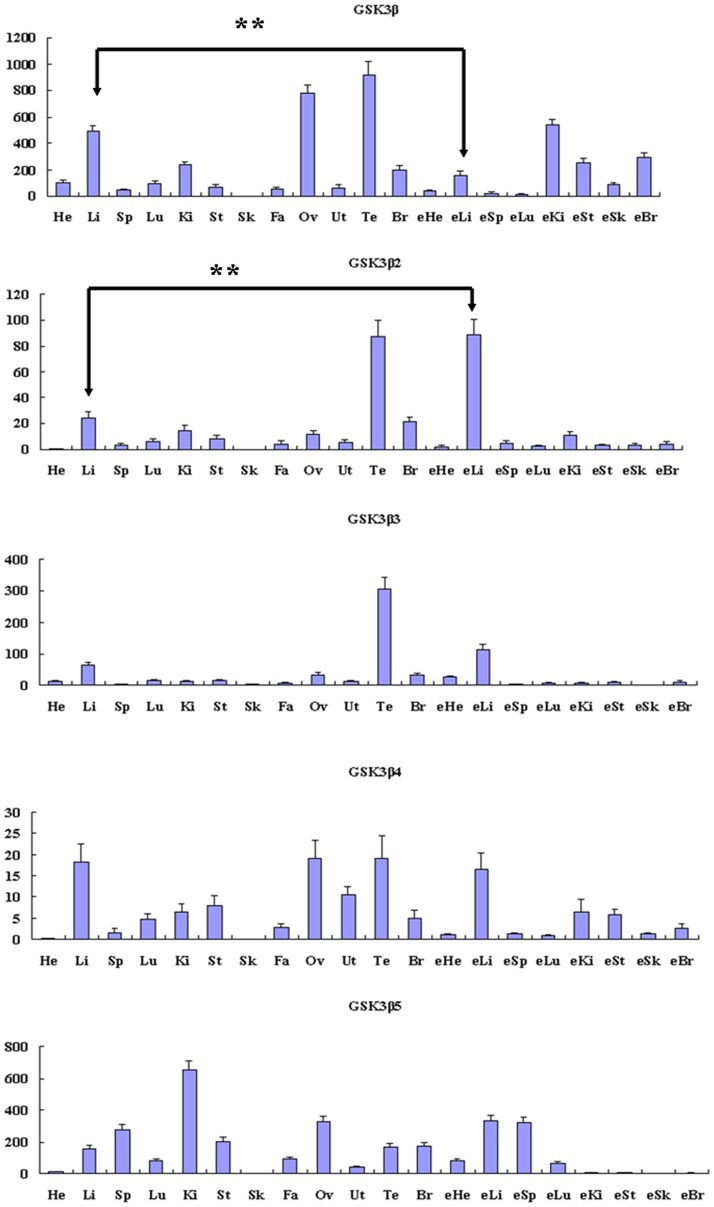
The tissue distribution of porcine GSK3β mRNA was assessed by quantitative real-time PCR. Error bars indicate the SD (n = 3) of relative mRNA expression levels of GSK3β to beta-actin, determined by qRT-PCR. The values were normalized to endogenous beta-actin expression.

The expression patterns were further confirmed and quantitated using isoform specific primer pairs (Table S1) to selectively amplify each of the GSK3β isoforms by qRT-PCR (Fig. 4). The mRNA of GSK3β1 was expressed in all twenty tissues examined, with the high level in adult liver, ovary, testis and fetal kidney. GSK3β2 and GSK3β3 have similar expression pattern with the high level in adult testis and fetal liver, whereas expressions in other tissues were relatively weak. The GSK3β4 was abundantly expressed in adult liver, ovary, testis and fetal liver but not in adult heart and skeletal muscle. In adult spleen, kidney and testis, the expression patterns of GSK3β5 were different from those in other isoforms. We also found that the GSK3β5 was abundantly expressed in adult spleen and kidney, whereas expressions in testis were relatively weak. In testis, all transcripts of GSK3β mRNA were at the highest level except for GSK3β5. Interestingly, the mRNA abundance of GSK3β1 of adult liver was significantly higher (p<0.01) than that in embryo. However, the expression level of GSK3β2 was significantly lower (p<0.01) in adult liver than that in fetal liver. Porcine GSK3β5 was most abundant in adult kidney, and undetectable in fetal kidney. The tissue distribution of five GSK3β isoforms raises the possibility that these isoforms may play different functions in porcine various tissues.

### Immunohistochemistry

**Figure 5:**
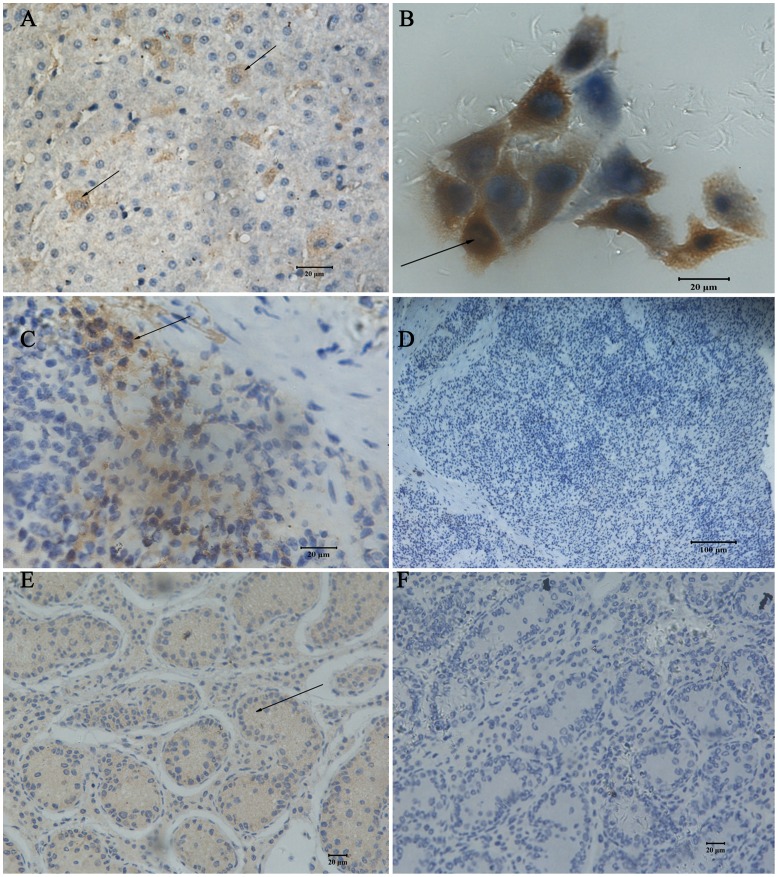
GSK3β protein expression in porcine tissues. Immunohistochemical analysis of porcine GSK3β using human anti-GSK3β in liver (A), spleen (C), testis (E) tissues and pig kidney epithelial cells (B) as compared with controls (D, and F). Arrows indicate positive staining.

Immunostaining performed on cross-sectional liver, spleen and testis cryosections using antibody directed against human GSK3β protein, porcine liver sections showed strong positive staining within the hepatocyte cytoplasm (Fig. 5A). The GSK3β protein was present in the nucleus of Sertoli cells, spermatogonia and spermatocytes in adult porcine testis (Fig. 5C). In the spleen, although porcine GSK3β can be seen in white pulp, GSK3β immunoreactivity was mainly detected in the red pulp (Fig. 5E). In pig kidney epithelial cells, porcine GSK3β was detected in the nuclei and cytoplasm.

### Effect of Insulin on Regulation of Different Porcine GSK3β Isoforms

**Figure 6:**
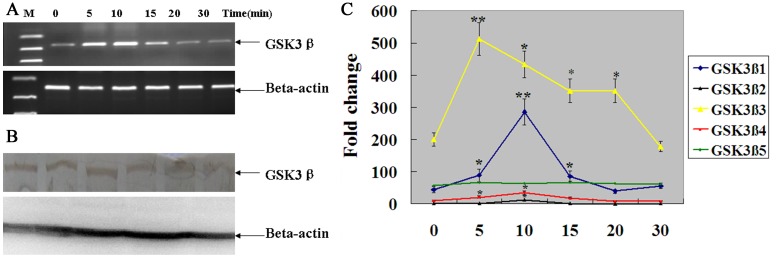
Differential time requirement for the change in mRNA expression level of different GSK3β isoforms in response to treatment with insulin. (A) Total GSK3β mRNA and protein expression was detected by semi-quantitative RT-PCR and western blotting, and Beta-actin was used as a control. (B) PK-15 cells were incubated in 100 nM insulin for 0, 5, 10, 15, 20 or 30 min after which cells total RNA was extracted for determining the expression of different GSK3β isoform by qRT-PCR. The values were normalized to endogenous beta-actin expression and the value of GSK3β2 after insulin incubation in 30 min was arbitrarily set to 1. Significant levels were analyzed by T-test. **, *p* < 0.01; *, *p* < 0.05.

To determine whether insulin affect the expression patterns of the GSK3β transcript variants, PK15 cells was treated for 0, 5, 10, 15, 20 or 30 min with 100 nM insulin. As shown in Fig. 6A, the porcine total GSK3β mRNA was up-regulated from the 0 min to 10 min after induction, reaching its highest expression at 10 min (p<0.01) and then declined. However, insulin did not significantly affect the total GSK3β protein level (Fig. 6B). In addition, the expression level of different GSK3β isoform is differentially regulated during the course of the insulin treatment (Fig. 6C). GSK3β1, GSK3β2 and GSK3β4 expression level was up-regulated from the 0 min to 10 min after induction, reaching its highest expression at 10 min (p<0.01) and then declined. Although the expression patterns of GSK3β1, GSK3β2 and GSK3β4 were similar, the mRNA expression level of GSK3β2 and GSK3β4 was much lower than that of GSK3β1. Moreover, porcine GSK3β3 was up-regulated at an earlier time following insulin treatment, increased to a peak at the 5 min (p<0.01), and then was down-regulated from the 5 min to 30 min after induction. No significant differences were detected in transcription of GSK3β5 during the course of the insulin treatment. These results suggest that GSK3β splicing variants may have different roles in insulin signaling pathway or others signaling pathways in pig through their different gene expressions.

### GSK3β5 have a Different Role on Regulating the Glycogen Synthase Gene Expression

To evaluate the effective overexpression of different GSK3β isoforms, whole cell lysates were harvested for mRNA extraction. All four constructs were overexpressed roughly 10-fold (Fig. S1) after 48 h transfection (with the exception of the GSK3β4, the constuction of pcDNA3.1-GSK3β4 was failed). Moreover, cells were transfected with pEGFP-GSK3β1, fluorescence detection revealed that 80–90% of the cells expressed GSK3β1 (Fig. S1).

**Figure 7:**
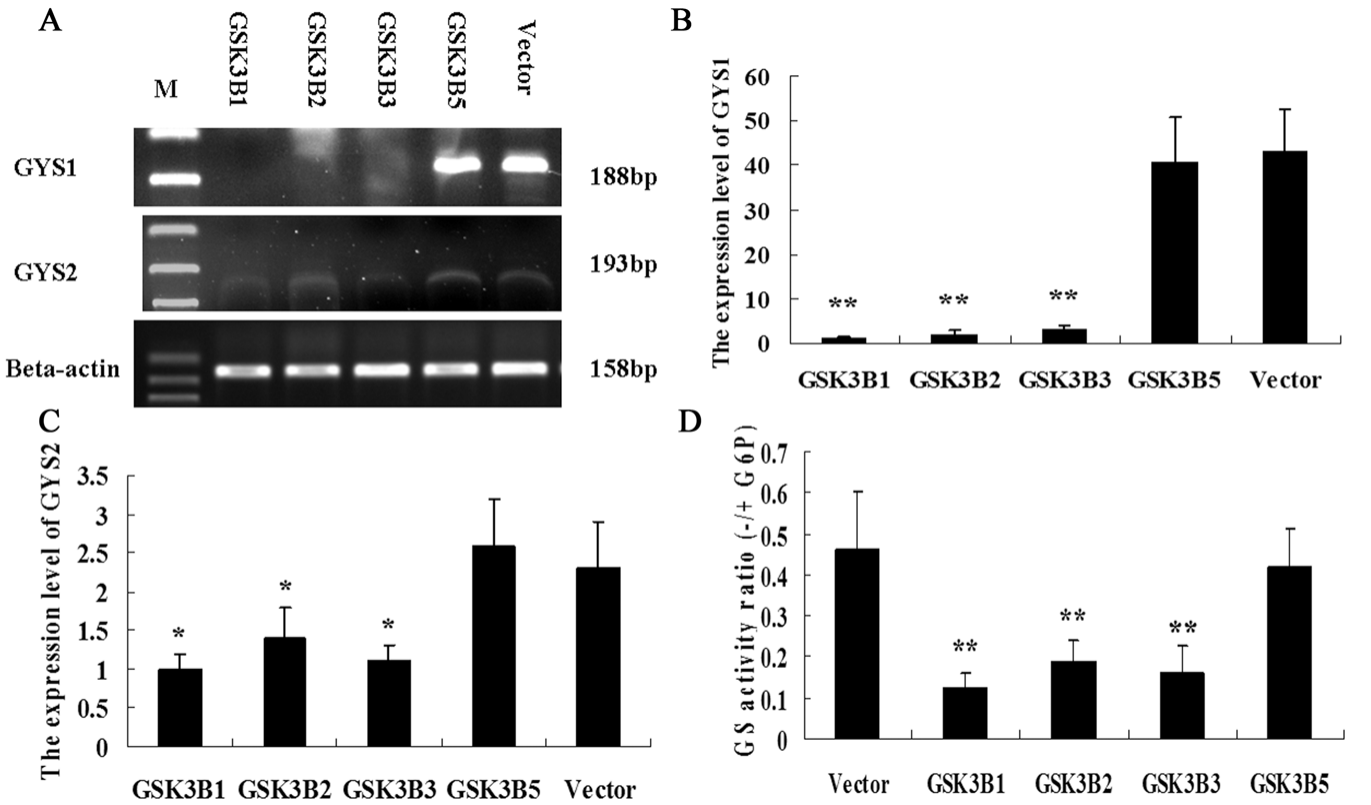
Effects of different GSK3β isoforms overexprssion on glycogen synthase activity. (A) Semi-quantitative RT-PCR analysis and Beta-actin was used as a control. (B, C) Quantitative real-time PCR was performed. The expression level of GYS1 and GYS2 was normalized to endogenous Beta-actin mRNA levels. Significant levels were analyzed by T-test. Data are expressed as means±SEM of three separate experiments. (D) PK-15 cells were tranfected in different GSK3β isoforms for the 48 h after which cells were lysed and we measured glycogen synthase activity in cells by assaying the incorporation of radioactive UDP-glucose into glycogen in the presence or absence of the allosteric activator glucose 6-phosphate (G6P).

In order to clarify the role of different GSK3β isoform on glycogen synthase in PK-15 cells, the expression level of porcine GYS1 and GYS2 gene encoding glycogen synthase was detected. We found that GSK3β5 had a different role on the expression level of porcine GYS1 and GYS2 gene. Compared to vehicle-transfected cells (Vector), overexpression of GSK3β1, GSK3β2 and GSK3β3 significantly reduced GYS1 (p<0.01) and GYS2 (p<0.05) mRNA expression level (Fig. 7A,B,C). However, the mRNA level of GYS1 and GYS2 was not significant change in cells overexpressing GSK3β5. No significant expression differences among cells overexpressing GSK3β1, GSK3β2 and GSK3β3 were observed.

### Regulation of Glycogen Synthase Phosphorylation and Activity Following Over-expression of Different GSK3β Isoforms

**Figure 8:**
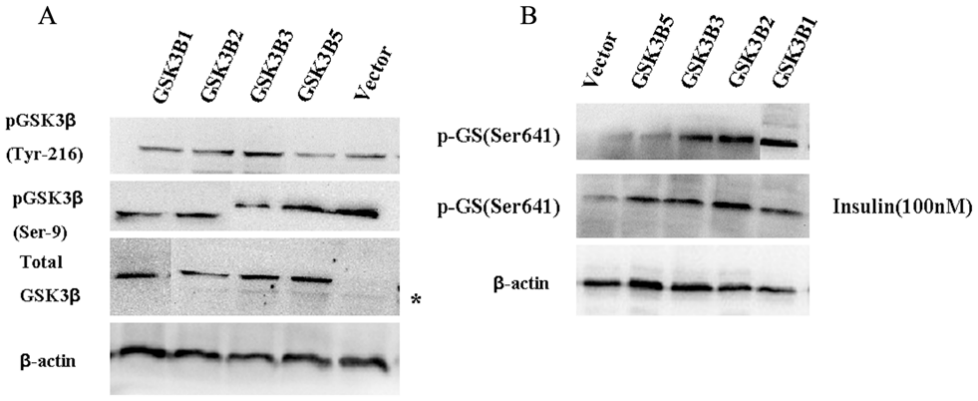
Regulation of glycogen synthase phosphorylation and activity following over-expression of different GSK3β isoforms. (A) Phosphorylation of pGSK3β (Ser9) and dephosphorylation of pGSK3β (Tyr216) levels in PK-15 cells transfected with different GSK3β isoforms. Equal amounts of lysate protein from cells were immunoblotted with antibody against phospho-Ser9-GSK3β, phospho-Tyr216-GSK3β and total GSK3β protein. *: Endogenous GSK3β. The blots are representative from up to three separate experiments. β-actin was used as the loading control. (B) Regulation of glycogen synthase phosphorylation following expression of different GSK3β isoforms induced by insulin (100 nM). PK-15 cells transiently transfected with pcDNA3.1-GSK3β1, 2, 3, 5 or the empty pcDNA3.1 expression vector. 24 h post-infection cells were incubated in the absence or presence of 100 nM insulin for 10 min. Following these incubations, cells were lysed and immunoblotting with antibodies against phosphor-Ser641-GS. β-actin was used as the loading control.

To define the activity of GSK3β isoforms in vitro, different GSK3β isoforms were significantly overexpressed in PK-15 cells. Then we assessed the phosphorylation level of GSK3β in vitro. As shown in Fig. 8A, Increased serine 9 phosphorylation and decreased tyrosine 216 phosphorylation were observed in PK-15 cells transfected with GSK3β1, GSK3β2 or GSK3β3, while over-expression of GSK3β5 caused a decrease in serine 9 phosphorylation levels of GSK3β, respectively.

Since glycogen synthase is a direct substrate of GSK3β, we examined its phosphorylation status and activity in cells expressing different GSK3β isoforms in absence or presence of insulin treatment. As shown in Fig. 8B, overexpression of GSK3β5 did not cause phosphorylation of glycogen synthase in PK-15 cells compared the control cells. Conversely overexpression of GSK3β1, 2 or 3 caused more phosphorylation of glycogen synthase (Fig. 8B). Moreover, the treatment with 100 nM insulin significantly reduced the phosphorylation level of glycogen synthase in PK-15 cells transfected with GSK3β1, GSK3β2 or GSK3β3, demonstrating the reduced activity of GSK 3β. These data show that increase in Ser9 phosphorylation of GSK 3β correlates with inhibition of GSK-3 kinase activity.

We further examined the effect of different GSK3β isoform on glycogen synthase activity by assaying the incorporation of radioactive UDP-glucose into glycogen in the presence or absence of the allosteric activator glucose 6-phosphate (G6P). As shown in Fig. 7D, overexpression of GSK3β1, GSK3β2, GSK3β3, but not GSK3β5, decreased significantly (p<0.01) glycogen synthase enzyme activity in PK-15 cells.

### Subcellular Localization of Porcine GSK3β Isoforms

**Figure 9:**
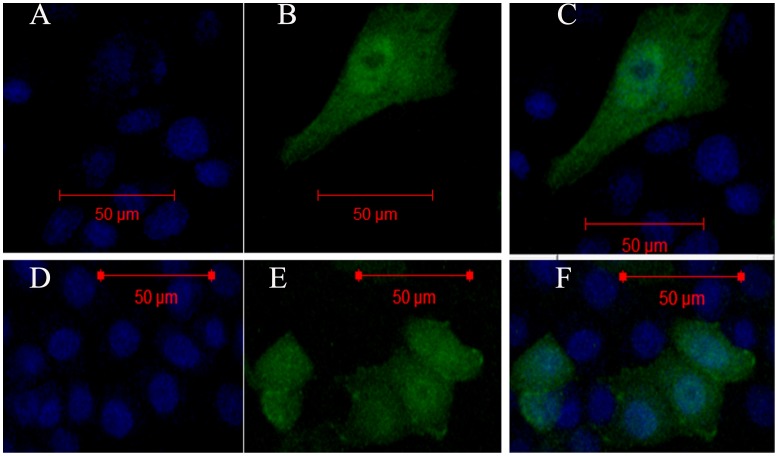
The subcellular localization of porcine GSK3β1 and GSK3β5 proteins in PK15 cells. The recombinant plasmid pEGFP-GSK3β1 and GSK3β5 were transiently transfected into PK15 cells using the Lipofectamine 2000 reagent. The GSK3β1 and GSK3β5-GFP fusion proteins were all distributed in localize both in cytoplasm and nuclei (excited at 488 nm; B, E), and nuclei were stained with DAPI (excited at 360 nm; A, D). The fluorescent signals were analyzed by confocal microscopy. The overlay images were produced by merging all three signals together (C, F).

The cellular locations of GSK3β1 and GSK3β5 isoforms were determined by fluorescence and confocal analysis of PK15 cells transiently transfected with pGFP-GSK3β1 and pGFP-GSK3β5 respectively. After labeling nuclei by staining with DAPI, these GFP fusion proteins were all found to localize both in cytoplasm and nuclei, displayed a nucleocytoplasmic distribution (Fig. 9). There is no different cellular localization among GSK3β1, GSK3β2, GSK3β3 and GSK3β5 isoforms (data not shown).

## Discussion

Previous studies in the human and mouse showed that GSK3β consists two different isoforms, GSK3β1 which distributes in many organs and the minor long form (GSK3β2), which has a 13-residue insert in the kinase domain, is present in central nervous system [12], [13]. In the present study, we observed multiple alternative splicing events of the GSK3β that occurred in porcine different tissues. One kind of the splicing occurs in the kinase domain, which results in a 13 amino acid insert (GSK3β2) and a 20 amino acid extraction (GSK3β5) in the kinase domain. The other contains the identical serine/threonine kinase domain (GSK3β3 and GSK3β4), but vary in their C-terminal (Fig. 1).

Interestingly, GSK3β5 lacks a 20 amino acid (203–222) in the kinase domain inculding three important phosphorylation site Lys 205, Tyr 216, Tyr 220 (Fig. 1). The crystal structure of GSK-3β reveals that the activation loop (residues 200–226) runs along the surface of the substrate binding groove [21]. Phosphorylation of Tyr 216, located on the activation loop, increases the catalytic activity of GSK3β [22]. Previous studies also show that the activities of GSK3 are regulated negatively by serine phosphorylation, but positively by tyrosine phosphorylation. Activities of GSK3 are positively regulated by phosphorylation of tyrosine residues 279 and 216 for α and β isoforms, respectively, and negatively regulated by N-terminal serine phosphorylation (residue 21 and 9 for α and β, respectively) [23], [24]. Our results show that GSK3β5 may lack positively regulated activation and plays different functions in regulating the substrate compared other GSK3β isoforms. GSK3β3 and GSK3β4 do not present Ser 389, which has been proposed as an inhibitory domain (Fig. 1). p38 mitogen-activated protein kinase (MAPK) inactivates GSK3β by direct phosphorylation at Ser 389 site and p38 MAPK-mediated phosphorylation of GSK3β at Ser 389 is sufficient to inhibit GSK3β activity [25]. So, GSK3β3 and GSK3β4 may lack negatively regulated activation and p38 MAPK could not inhibit GSK3β activity through Ser 389. However, the physiological significances of these variants are unclear.

Insulin/IGF-1 stimulate glycogen and protein synthesis mainly mediated via the signaling cascade PI(3)K/Akt/GSK3β that leads to the phosphorylation of GSK3 and GSK3 kinase with inhibited activity [26]. This inactivation leads to a decrease in phosphorylation of GS and eIF2B resulting in its activation and promotes glycogen and protein synthesis [27]. In our previous study, insulin can promote phosphorylation of GSK3β and dephosphorylation of GS in differentiated porcine satellite cells, and insulin can promote the of activity GS [28]. In this study, we observed multiple alternative splicing events of the GSK3β, and the mRNA expression level of GSK3β isoforms is differentially regulated during the course of the insulin treatment, suggesting that different GSK3β isoforms may have different roles in insulin signaling pathway.

We found that GSK3β5 have no effect on the mRNA expression level of porcine GYS1 and GYS2 gene. However, overexpression of GSK3β1, GSK3β2 and GSK3β3 significantly reduced the mRNA expression level of GYS1 and GYS2 (Fig. 7). There are numerous transcription factors that have been proposed to be substrates for GSK-3 including CREB [29], NF-kappaB [30], AP-1 [31], β-catenin, c-jun, heat shock factor 1, p53, and Bax [32]. In our previous study, the porcine GYS1 and GYS2 promoter both contains several binding sites for transcription factors of the NF-kappaB (nuclear factor B), and CREB (cAMP responsive element binding) [28]. In addition, GSK-3α and GSK-3β have been shown to have differentially regulated transactivation in causing cAMP-responsive element and NF-kappaB-dependent transactivation [33]. So, we infer that GSK3β may regulate GYS1 and GYS2 gene transcription through the NF-kappaB and CREB element, and GSK3β5 may have different role on regulating the gene expression compare other GSK3β isforms.

When we examined the effect of different GSK3β isoform on glycogen synthase activity, found that overexpression of GSK3β1, GSK3β2, GSK3β3, but not GSK3β5, decreased significantly (p<0.01) glycogen synthase enzyme activity in PK-15 cells. Pervious studied showed that the inhibition of GSK3 promotes the dephosphorylation and activation of glycogen synthase, contributing to the stimulation of glycogen synthesis [34], [35]. GSK3 activity is significantly reduced by phosphorylation of Ser9 in GSK3β and Ser21 in GSK3α. In opposition to the inhibitory modulation of GSK3β that occurs by serine phosphorylation, tyrosine phosphorylation of GSK3β increases the enzyme’s activity, and its activity is dependent on tyrosine phosphorylation on Tyr-216 [23], [24], [36]. In the present study, we detected a novel GSK3β isoform, named GSK3β5, which lacks a 20 amino acid (203–222) in the kinase domain including three important phosphorylation site Lys 205, Tyr 216, Tyr 220. So we infer that GSK3β5 lack the GSK3 kinase positively regulated activity and have no effect on glycogen synthase enzyme activity.

The subcellular distribution of GSK3β regulates its actions by controlling its accessibility to substrates, such as many transcription factors in the nucleus [20]. In this study, we found that GSK3β5 have a different role on the mRNA expression level of porcine GYS1 and GYS2 gene. So we further examined the cellular locations of each GSK3β isoform. These GSK3β-GFP fusion proteins were all found to localize both in cytoplasm and nuclei, displayed a nucleocytoplasmic distribution. Previous study revealed that the nuclear distribution of GSK3β is regulated by the NLS locating in 85–103 residues of N-terminal [37]. The five different GSK3β isoforms both contain the nuclear localization sequence (NLS). Nuclear GSK3β levels decrease, when treated by proliferative growth factors [38]. In addition, nuclear export of GSK3β is partially mediated by FRAT-1, which binds GSK3β in the nucleus followed by export of the complex via the nuclear export sequence (NES) in FRAT-1 [39], [40]. So, the more precise localizations of them in various conditions require further investigations to help us deeply understand their different biological functions. Determination of the unique properties, possibly protein partners of GSK3β isoforms will likely provide valuable insight into the special actions of their substrates.

## Materials and Methods

### Ethics Statement

All research involving animals were conducted according to the regulation (No. 5 proclaim of the Standing Committee of Hubei People’s Congress) approved by the Standing Committee of Hubei People’s Congress, P. R. China. Sample collection was approved by the ethics committee of Huazhong Agricultural University (No. 30700571 for this study).

### RNA Isolation and cDNA Synthesis

Adult Meishan pigs (females, four-month-old, n = 3; males, four-month-old, n = 3) and pig embryos [65day post conception (dpc), n = 3] were all obtained from Jingpin Pig Station of Huazhong Agricultural University (Wuhan, China). Porcine adult heart, liver, spleen, lung, kidney, stomach, longissimus dorsi muscle, subcutaneous adipose tissue, ovary, uterus, testis, brain, and embryo heart, liver, spleen, lung, kidney, stomach, longissimus dorsi muscle and brain were freshly collected and then immediately frozen in liquid nitrogen pending RNA extraction. Male mouse (kunmingbai, two-week-old, n = 3) were purchased from a supplier (Chengdu Center for Disease Prevention and Control, Sichuan, China). Mouse heart, liver, kidney, testis, brain and skeletal muscle were also collected. The RNA was extracted using Trizol reagent (Invitrogen) according to the manufacturer’s protocol, treated with RNase-free DNase I (Takara, Japan) to remove contaminating genomic DNA and stored at −80°C. The first strand cDNAs was synthesized using M-MLV reverse transcriptase (Promega, Madison, WI, USA) as described in protocol. The corresponding cDNA was stored at –20°C.

### In Silico Cloning of Porcine GSK3α and GSK3β Gene

Human cDNA sequences of *GSK3α* and *GSK3β* (GenBank accession no. NM_019884.2 and NM_002093.3) were compared to all sequences available in the pig EST databases using the BLAST algorithm (http://www.ncbi.nlm.nih.gov/blast). We selected the porcine ESTs that shared more than 85% sequence identity to the corresponding human cDNA to assemble the porcine *GSK3α* and *GSK3β* gene using the DNA Star program (Madison, WI, USA). Two primer pairs (CDS-AF, CDS-AR for GSK3α and CDS-BF, CDS-BR for GSK3β, Table S1) in both 5*′* and 3*′* untranslated region covering the entire coding sequences were designed to PCR amplify coding regions of porcine *GSK3α* and *GSK3β* gene.

### Detection of Splice Forms of GSK3β

For identification of splice forms of porcine *GSK3β*, RT-PCR was carried out using porcine adult liver, testis, brain cDNA and porcine fetal liver, testis, brain cDNA using the primer pairs CDS-BF and CDS-BR. The PCR products were separated by 2.0% agarose gel electrophoresis, the all PCR bands were cut out of the agarose gel and purified using a Gel Extraction Kit (Sangon, Shanghai, China). The purified products were then sub-cloned into the pMD-18T vector (Takara, Japan). Plasmids randomly isolated from individual colonies from porcine adult liver, testis, brain cDNA and fetal liver, testis, brain cDNA. The sequences were analyzed by Beijing AuGCT Biotechnology Company.

NCBI’s online ORF Finder (http://www.ncbi.nlm.nih.gov/gorf/gorf.html) and DNASTAR were employed to predict open reading frames for translated peptide products. We analyzed the physico-chemical parameters of the deduced protein sequence employing ProtParam (http://cn.expasy.org/tools/protparam.html). Bioinformatics domain searching analysis was performed using PROSITE (http://au.expasy.org/prosite/). Furthermore, ClustalW (http://www.ebi.ac.uk/clustalw/) was used for multiple sequence alignment and shaded using BOXSHADE 3.21 (http://www.ch.embnet.org/).

### Reverse Transcription-PCR

Reverse transcription-PCR (non-quantification) was used to amplify individual isoforms of *GSK3β* from the cDNAs of different adult and fetal tissues of Meishan pigs. PCR cycling conditions were as follows: 95°C initial denaturation for 4 min, 35 cycles of 95°C denaturation for 40 s, 60°C annealing for 40 s, and 72°C extension for 20 s. A final extension was performed at 72°C for 7 min. The PCR fragments were purified and directly sequenced to confirm the correct amplification of the individual isoforms. For the specific amplification of each *GSK3β* cDNA isoform, five new primer pairs were used (Table S1); GSK3B-V2F/2R in exon8 and exon8 b, GSK3B-V3F/3R in exon10 b and exon11, GSK3B-V4F/4R located at exon8 and the junction of exon9 and exon11, and GSK3B-V5F/5R located at the junction of exon6, exon7 and exon9. Primer pairs GSK3B-V1F/1R located at the junction of exon8, exon9 and the junction of exon10, exon11 to amplify the *GSK3β1* and *GSK3β5* (Fig. 1). Beta-actin was used as an endogenous reference gene.

### Quantitative Real Time RT-PCR Analysis

Real-time RT-PCR was used to quantify the expression level of porcine GSK3β isoforms in different adult and fetal tissues using ABI 7300 real-time PCR thermal cycle instrument (ABI, USA), according to the supplied protocol. Each real-time PCR (in 25 µL) reaction contained 12.5 µL SYBR® Green Real time PCR Master Mixture (contains ROX Dye. Toyobo, Jap), 0.25 µM primers and 1 µL normalized template cDNA. The cycling conditions consisted of an initial, single cycle for 3 min at 95°C followed by 40 cycles of cycling consisting of 20 s at 94°C, 20 s at 60°C, 15 s at 72°C, and final extension for 5 min. The primers were the same as those mentioned in the RT-PCR. The specificity of PCR products were confirmed by melting curve analysis. Pooled cDNA from a subset of the liver samples examined in this study was used to generate the standard curves. In this assay, the efficiency of Beta-actin, GSK3β1, GSK3β2, GSK3β3, GSK3β4 and GSK3β5 gene primers were 96.5%, 97.5% 97.1%, 96.2%, 98.2%and 97.7%, respectively. The amplification efficiencies of control and target genes are approximately equal. Gene expression levels were quantified relative to the expression of Beta-actin using Gene Expression Macro software (ABI, USA) by employing an optimized comparative Ct (2-ΔΔCt) value method. All PCR amplifications were performed in triplicate for each RNA sample.

### Cryosection and Immunohistochemistry

Immunohistochemical examination was undertaken on liver, slpeen and testis samples from three male Meishan pigs. Tissues were embedded in OCT medium (Tissue Tek, Miles, Elkhart, IN, USA) and frozen at −20°C, and serial 5-µm sections were cut with a cryostat (Leica, Bensheim, Germany). Slides were incubated using anti-GSK3β (1∶200) at 4°C overnight. Secondary antibody was horseradish peroxidase-conjugated goat anti-rabbit IgG (1∶2000) and was incubated for 30 min. The SABC and DAB visualization methods were used according to the manufacturer’s instructions (Boster Company, China). The sections were counter-stained with hematoxylin for 10 min. Negative controls were done by omitting the primary antibody or by using an irrelevant primary antibody of the same isotype. Stained muscle cross-sections were viewed with an Olympus BX-50F light microscope (Olympus Optical, Tokyo, Japan).

### Time-course Effect of Insulin in PK-15 Cells

PK15 cells were plated in 6-well plates at a concentration of 2.5×10^5^ cell/well (2 mL/well) and cultured in Dulbecco’s modified Eagle’s medium (DMEM) supplemented 10% (v/v) fetal bovine serum under humidified air containing 5% CO2 at 37°C. To evaluate the time-dependent changes in the levels of each GSK3β isoform, when 70%–80% confluence was observed, PK-15 cells were serum starved for 6 h, then 100 nM bovine insulin (Sigma, Cat. Nr: I0516) was added for 0, 5, 10, 15, 20 or 30 min. Total RNA and protein was extracted for determining the expression of different GSK3β isoform by qRT-PCR and western blotting, respectively.

### Plasmid Construction and Transfection

To construct porcine different GSK3β isoform recombinant plasmids for expression in mammalian cells, the open reading frames coding for different porcine GSK3β isoforms were amplified from cDNAs of porcine liver using primer pair (GSK3B-GFPF, GSK3B-GFPR, Table1). The PCR products were first cloned into a pGEMT easy vector (Promega), then digested with *Kpn*I and *Xho*I enzymes for sub-cloning into the pcDNA3.1(+) and pEGFP-N1 vector to generate the pcDNA3.1-GSK3β and pEGFP-GSK3β plasmids. Correct orientation and reading frame were confirmed by sequencing.

PK15 cells were cultured as mentioned above. Transient transfection was performed using lipofectamine 2000 (Invitrogen) according to the manufacturer's instructions, and then cultured in media an additional 48 h before mRNA extraction or assay of glucose uptake. For detecting the cellular locations of each GSK3β isoform, At 24 h after transfection, cells were washed three times with phosphate-buffered saline (PBS), and then fixed in pre-warmed growth medium containing 4% formaldehyde for 15 min at 37°C. After the final washing steps and incubation with 10 µM DAPI for 10 min, the slides were mounted and sealed, and analyzed by confocal microscopy (TCS-SP2). Leica confocal software (Leica IM500) was used to generate images of individual fluorescent markers as well as overlay pictures to demonstrate the relative distribution of the fusion protein.

### Western Blotting

Total protein was extracted in preparation buffer [7 M urea, 2 M thiourea, 4% CHAPS, 1% DTT, 2% IPG Buffer pH3–10, 10 µl proteinase inhibitor cocktail (BBI, Kitchener, Canada)]. Then incubated for 30 min at room temperature with occasional vortex, and centrifuged at 20,000 g for 15 min at 4. The supernatant was collected and stored at -80 until analysis. Protein concentrations were determined using the Bradford protein assay. 20 µg of total protein extract from PK-15 cells was separated by 10% SDS-PAGE and subsequently electro transferred to PVDF membrane, and incubated with antibodies to phospho-Ser9-GSK3β (Santa Cruz, CA) and phospho-Tyr216-GSK3β (Santa Cruz, CA) at a 1∶400 dilution; GSK3β (Cell signaling, Danvers, MA) and phospho-Ser641-GS (Cell signaling, Danvers, MA) at a 1∶1,000 dilution at 4C overnight. Immunoblots were developed using horseradish peroxidase-conjugated goat anti-rabbit IgG (Santa Cruz, CA) at a 1∶5,000 dilution, followed by detection with enhanced chemiluminescence.

### Glycogen Synthase Assay

PK-15 cells were transfected with different pcDNA3.1-GSK3β plasmids for 24 h then scraped into 600 µl of homogenization buffer (10 mM Tris-HCl (pH 7.8), 150 mM NaF, 15 mM EDTA, 60 mM sucrose, 10 µg/ml leupeptin, and 50 mM sucrose, 1 mM PMSF). Samples were centrifuged at 4°C and 12000 g for 10 min and the supernatant was saved for GS assay. GS activity was assayed by measuring the incorporation of glucose from UDP-[U-^14^C] glucose into glycogen [28]. Briefly, assay buffer (50 mM Tris-HCI, pH 7.8, 20 mM EDTA, 25 mM KF, 1% glycogen, 200 UDP-[U-14C] glucose (4.5 mCi/umol) was added to 45 µL cell lysate in the presence and absence of 20 mM glucose-6-phosphate. After a 30 min incubation at 37°C the reaction was stopped by spotting the reaction mix onto Whatman No.5 filter paper and washed three times in 66% (v/v) ethanol for 20 min. Filters were washed in acetone for 5 min and dried before being counted in a liquid scintillation counter. Glycogen synthase activity was expressed as a ratio of activity in the absence divided by that in the presence of its allosteric activator, glucose-6-phosphate.

## Supporting Information

Figure S1 **The effect of GSK3β isoforms overexpression.** PK-15 cells were transfected with pcDNA3.1-GSK3β1, GSK3β2, GSK3β3, GSK3β5, respectively. Cells were harvested for mRNA extraction after 48 hours transfection. (A) The effective overexpression of different GSK3β isoforms, was identified by Semi-quantitative RT-PCR and Beta-actin was used as a control. (B) PK-15 cells were transfected with pEGFP-GSK3β1. Cells were fixed and analyzed by immunofluorescence after 48 hours transfection.
(TIF)

Table S1 **Primer sequences used in this study.**
(DOC)
