# Supplementary figures and images for: Alternative Splicing of the Porcine Glycogen Synthase Kinase 3β (GSK-3β) Gene with Differential Expression Patterns and Regulatory Functions
Source: PLoS One. 2012 Jul 6;7(7):e40250. doi: 10.1371/journal.pone.0040250 (PMC3391277; doi:10.1371/journal.pone.0040250)

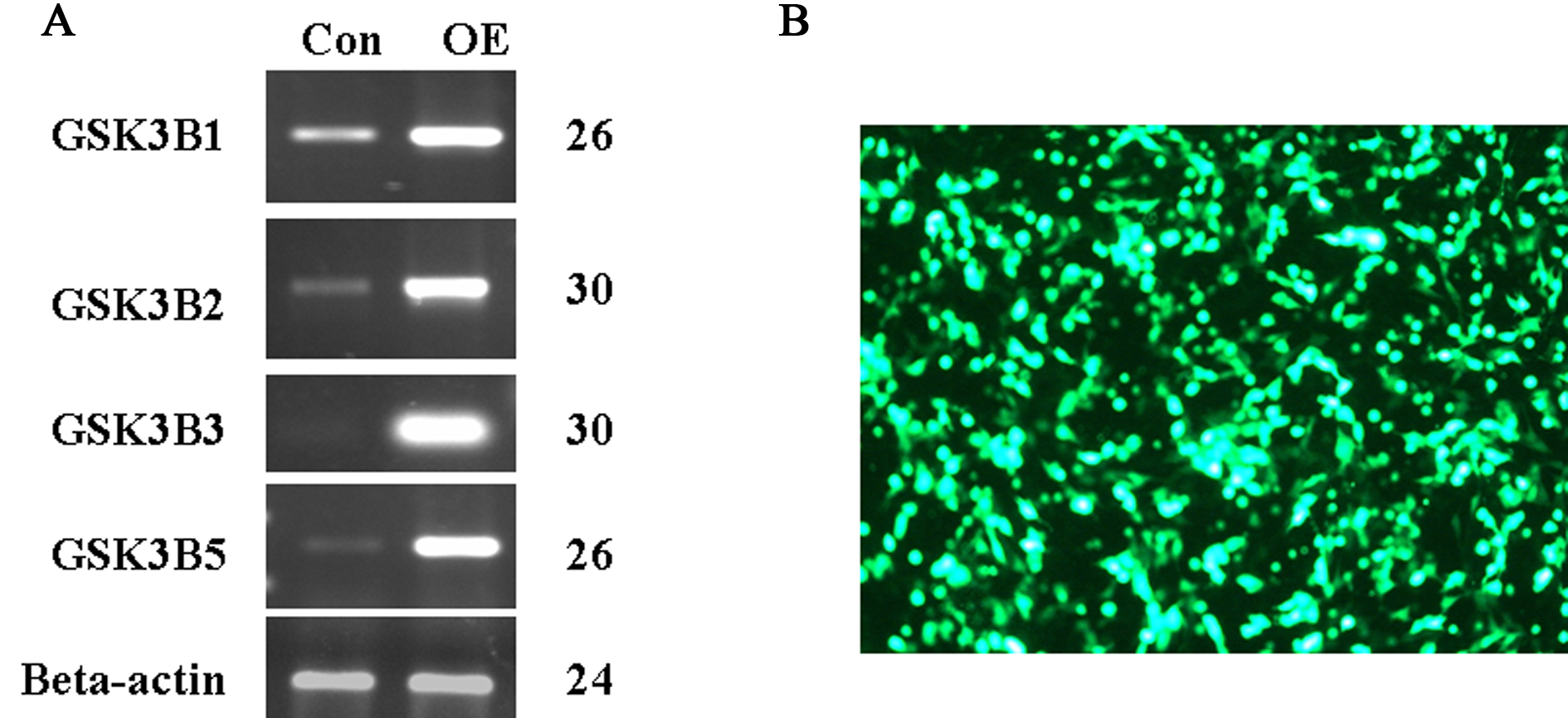

Supplement: Figure S1 — The effect of GSK3β isoforms overexpression. PK-15 cells were transfected with pcDNA3.1-GSK3β1, GSK3β2, GSK3β3, GSK3β5, respectively. Cells were harvested for mRNA extraction after 48 hours transfection. (A) The effective overexpression of different GSK3β isoforms, was identified by Semi-quantitative RT-PCR and Beta-actin was used as a control. (B) PK-15 cells were transfected with pEGFP-GSK3β1. Cells were fixed and analyzed by immunofluorescence after 48 hours transfection. (TIF) [file pone.0040250.s001.tif]
