# Supplementary material for: Alternative Splicing of the Porcine Glycogen Synthase Kinase 3β (GSK-3β) Gene with Differential Expression Patterns and Regulatory Functions
Source: PLoS One. 2012 Jul 6;7(7):e40250. doi: 10.1371/journal.pone.0040250 (PMC3391277; doi:10.1371/journal.pone.0040250)
Supplement: Table S1 — Primer sequences used in this study. (DOC) [file pone.0040250.s002.doc]

Table S1: Primer sequences used in this study

| Gene name | Primer name | Primer sequence (5′–3′) | Binding region | Size(bp) | Tm (°C) |
| --- | --- | --- | --- | --- | --- |
| GSK3α | CDS-AF  CDS-AR | GCTCGGCGCCATGAGCGG  GCAGGTTTTCATGCCAATA | 5'-UTR  3'-UTR | 2064 | 64 |
| GSK3β | CDS-BF  CDS-BR | CCATTTCTACCCTCCAAG  TAACTGGTGGTTCTTCCT | 5'-UTR  3'-UTR | 1599 | 58 |
|  | GSK3B-V1F  GSK3B-V1R | CCTTGGACTAAGGTCTTCC  GGCATTAGTATCTGAGGCT | Exon8-Exon9  Exon10-Exon11 | 307 | 61 |
|  | GSK3B-V2F  GSK3B-V2R | CACCAACAAGGGAGCAAAT  CGCACTCCTGAGGTGAAAT | Exon8  Exon8b | 124 | 60 |
|  | GSK3B-V3F  GSK3B-V3R | CCCTTCTAACAGAAAAGGGAA  TCAGGTGGAATTGGAAGCTG | Exon10b  Exon11 | 110 | 60 |
|  | GSK3B-V4F  GSK3B-V4R | AGGCACATCCTTGGACTAAG  CCGGCATTAGTATCTTGAGT | Exon9  Exon10-Exon11 | 220 | 60 |
|  | GSK3B-V5F  GSK3B-V5R | GACTTTGGAAGGGCACCAGA  CCTTGTTGGTGTCCCTAGGA | Exon5-Exon6  Exon8 | 177 | 60 |
|  | GSK3B-GFPF  GSK3B-GFPR | CGCTCGAGATGTCAGGGCGGCCCAGAA  GGGGTACCGGTGGAATTGGAAGCTGACG | Exon1  Exon11 | 1266 | 62 |
| GYS1 | GYS1RT-F  GYS1RT-R | GCCGACAGGGTCAAGGTAA  CGGAGAGGTTGGTGGAGAT | Exon 11  Exon 13 | 190 | 60 |
| GYS2 | GYS2RT-F  GYS2RT-R | CATCACCACCAACGACGGA  ACACGGCCCAGAGAAAAGG | Exon 15  Exon 15 | 193 | 60 |
| Beta-actin | Actb-F  Actb-R | GGTCAAGCAGCATAATCCAAAG  CAAGGGCATAGCCTACCACAA |  | 158 | 60 |
